# The barriers, motives, perceptions, and attitudes toward research among radiology practitioners and interns in Saudi Arabia: a cross-sectional study

**DOI:** 10.3389/fmed.2023.1266285

**Published:** 2023-10-09

**Authors:** Khalid M. Alshamrani, Abdulkader A. Alkenawi, Reham E. Kaifi, Khaled A. Alhulil, Wael M. Munshi, Abdulaziz F. Alqurayqiri, Faisal A. Alshehri, Hawazen I. Abdulmannan, Enas M. Ghulam, Sameer E. Tasslaq, Ali M. Aldhebaib

**Affiliations:** ^1^College of Applied Medical Sciences, King Saud bin Abdulaziz University for Health Sciences, Jeddah, Saudi Arabia; ^2^King Abdullah International Medical Research Center, Jeddah, Saudi Arabia; ^3^Ministry of the National Guard—Health Affairs, Jeddah, Saudi Arabia; ^4^College of Science and Health Professions, King Saud bin Abdulaziz University for Health Sciences, Jeddah, Saudi Arabia; ^5^College of Applied Medical Sciences, King Saud bin Abdulaziz University for Health Sciences, Al-Ahsa, Saudi Arabia; ^6^College of Applied Medical Sciences, King Saud bin Abdulaziz University for Health Sciences, Riyadh, Saudi Arabia

**Keywords:** radiologic technology, radiologic research, research culture, healthcare professions, Kingdom of Saudi Arabia

## Abstract

**Background:**

Research and the use of evidence-based practices are imperative to the advancement of diagnostic imaging modalities. The aim of this study was to assess the perceptions and attitudes of radiology practitioners (i.e., Technicians, Technologists or Specialists, and Senior Specialists) and interns in King Abdulaziz Medical Cities (KAMCs), Kingdom of Saudi Arabia, toward research, and to explore the various barriers and obstacles that hinder their research efforts.

**Methods:**

A cross-sectional descriptive investigation was carried out from December 2022 to March 2023 among 112-KAMCs’ radiology practitioners and interns, using previously developed and validated questionnaire comprised of five distinct sections, each serving a specific purpose, and with a non-probability convenient sampling technique. Descriptive statistics were generated for participants’ demographics, and chi-square and fisher’s exact tests were used to examine the association between participants’ demographics and their involvement in research.

**Results:**

Among the 137 KAMCs’ radiology practitioners and interns who were invited to participate, 112 responded and completed the questionnaire, resulting in an overall response rate of 81.75%. Radiology practitioners and interns from various medical imaging subspecialties were found to be involved in research to the extent of 83%, with nearly half (40.9%) of them have had publications, and 53.3% of these publications being either cross-sectional studies or retrospective clinical studies. A lack of time (66.1%), a lack of a professional supervisor support program (50.9%), and deficiency in research skills (45.5%) were common obstacles that may impede the participants’ ability to conduct research. The most common motives for participants to conduct research were the desire to improve their resumes (69.6%), get accepted into postgraduate radiology programs (58%), and improve their research skills (52.7%).

**Conclusion:**

KAMCs’ radiology practitioners and interns have a positive attitude toward performing research. Despite the high percentage (83%) of those involved in research, the number of publications remains low. A crucial step to advancing the profession’s evidence base is engaging radiology practitioners and interns in research and encouraging radiology practitioner-led research. The study findings can serve as a valuable basis for designing developmental programs aimed at overcoming research obstacles among healthcare professionals in Saudi Arabia.

## Introduction

The advancement of diagnostic imaging modalities is contingent upon the utilization of evidence-based practice (EBP), and the active participation in research (1–6). Enhancement of research competencies is necessary both at the personal and organizational levels in order to achieve a lasting advancement in the field of health research (7). Several approaches are being utilized by educational and health institutions to motivate individuals to undertake careers in research, such as obligatory and elective research tasks, dedicated student sections in prominent journals, establishment of student-led scientific conferences, incorporation of research capacity building within applied health sciences’ curriculum, and facilitation of workshops on diverse aspects of research methodology (8).

Radiologists and physicists have a long history of conducting radiation science research, and despite being in the early stages of radiologic research, radiographers’ academic progression continues as they perform research studies in addition to their university teaching responsibilities (1, 9–11). However, a distinction should be made between medically trained professionals such as Radiologists, and physicists compared to radiographers, as their distinct curricula can impact their motivation for research. Enablers of undergraduate research mirror its constraints (12). These consist of curriculum strategies such as teaching on research methodology, providing early exposure to research experiences, and elective research activities (13–15); adequate resources and formal infrastructural support (14–19); enhancing supervision capacity and support (18–20), doing research in groups; (13, 21) and promoting and raising awareness about research opportunities while fostering a sense of community in a research setting by recognizing students’ research accomplishments (14–16, 19, 20, 22). Researchers in the field of radiography have investigated a variety of topics, including the advancement in medical imaging technology and its impact on patient well-being and quality of care (23, 24). Investigating clinical radiographers’ motive for conducting radiologic research has numerous compelling reasons. First and foremost, radiographers bear a significant responsibility for and possess unparalleled expertise in providing patient care in the field of diagnostic imaging (1). A second reason is the rapid and ongoing advancement of diagnostic imaging technology. Digital medical imaging and patient administration systems have advanced, hybrid imaging has been introduced, and artificial intelligence has become more widespread (25–28). Additionally, it is of paramount importance to emphasize on the critical role that radiology professionals play in ensuring patient radiation safety during medical imaging procedures. Radiology professionals must adhere to the “as low as reasonably achievable” (ALARA) principle by keeping occupational radiation dose and patient exposure as low as possible, while maintaining excellent image quality when preforming radiologic examination (29).

With the current technological advances and the crucial role radiographers play in linking technology to patients, clinical radiographers need to participate actively in radiologic research progress. The role of radiographers is no longer limited to keeping up with technology; they are also required to progress patient-centered developments and contribute to healthcare and technology research. This suggests a transformation of role from that of a clinical radiographer to that of a researcher (1).

A growing number of radiology practitioners are embracing research and utilizing research evidence in several countries, including Portugal, Denmark, Norway, Sweden, Singapore, and Finland (1, 2, 30–33). The number of publications in radiography has been significantly increased over the last decade (2, 34), but radiographers’ reported participation in research has remained relatively low. Additionally, those who have participated in research have acquired research experience during their academic studies (2, 30–33).

In the Kingdom of Saudi Arabia (KSA), research perceptions, barriers and attitudes have been reported among physicians, residents, and senior medical students (35–37). However, the attitudes and behavior of radiology practitioners and interns in Saudi Arabia with regards to research remain unknown due to a scarcity of empirical studies. To bridge this gap, our study seeks to determine the opinions of radiology practitioners and interns about radiologic research, as well as to investigate their participation in research activities. The specific aim of this study was to assess the perceptions and attitudes of radiology practitioners (i.e., Technicians, Technologists or Specialists, and Senior Specialists) and interns in King Abdulaziz Medical Cities (KAMCs), KSA toward research, while also exploring the various barriers and obstacles that hinder their research efforts. Additionally, the study explored factors that influence radiology practitioners’ and interns’ perception of the importance of research and the motivations. In the KSA, a Radiology Technologist or Specialist is a professional who has attained a bachelor’s degree, which includes 4 years of undergraduate studies followed by 1 year of internship, while a Radiology Technician is a professional who has attained a diploma certificate after completing 2 years of undergraduate studies (38). This study serves as an initial step toward increasing awareness among healthcare professionals and organizations in Saudi Arabia, as structured initiatives to improve clinical radiologic research capacity can be guided by assessing practitioners’ and interns’ interest in and motivation for research.

## Methods

### Study design and setting

A descriptive cross-sectional questionnaire study was carried out among radiology practitioners and interns from the medical imaging departments of KAMCs in Jeddah, Riyadh, and Al Ahsa in the Kingdom of Saudi Arabia. The study was conducted from December 2022 to March 2023.

### Study participants and sampling technique

Using a non-probability convenient sampling technique, an estimated population of 137 radiology practitioners and interns were invited to participate voluntarily in this study. The questionnaire was disseminated via both WhatsApp and email to radiology practitioners and interns employed across six distinct divisions/subspecialties of the medical imaging department [i.e., Radiography/Mammography/Fluoroscopy, Magnetic Resonance Imaging (MRI), Computed Tomography (CT), Nuclear Medicine, Ultrasonography, and Interventional Radiology].

### Data instruments

Participants were instructed to complete a previously used and validated questionnaire (36, 37). The questionnaire was reviewed by five doctorate of philosophy (PhD) degree holders/senior radiology practitioners with extensive experience in radiologic technology. The purpose of this review was to ensure that the questionnaire was specifically targeted toward the study radiology demographics, was clear and concise, and maintained a focused and purposeful approach. The questionnaire consisted of closed-ended multiple-choice questions that collected information about participants. The questionnaire was comprised of five distinct sections, each serving a specific purpose. The first section gathered socio-demographic information pertaining to the participants, including their age, gender, nationality, marital status, Grade Point Average (GPA), institutional affiliation, years of experience, and the country from which they obtained their Radiological Technologist degree. The second section explored any obstacles or barriers that may impede the participants’ ability to conduct research, taking into consideration both personal and institutional factors. In the third section, participants were asked to reflect on their prior experience and involvement in research work including the number and type of publications. The fourth section sought to gauge the perceptions of both radiology practitioners and interns regarding the importance of research and its potential impact on their professional careers. Finally, the fifth section was designed to explore the factors that motivate radiology practitioners and interns to conduct research.

### Ethical consideration

King Abdullah International Medical Research Center ethics committee approved this study (Study Number: SP22J/099/08).” The involvement of participants was completely voluntary, and written informed consent was obtained prior to the completion of the questionnaire. Anonymity and confidentiality were maintained throughout the study. This was specifically demonstrated by the use of a password-protected Microsoft Excel file which was exported from the electronic survey tool. The file did not reveal any subject identification attributes.

### Statistical analyses

The statistical analysis consisted of a three-step procedure. First, frequencies and percentages were computed for the demographics of the participants and their responses to the questionnaire items. Second, chi-square test was used to examine the association between participants’ demographics (i.e., gender, marital status) and participation in research (i.e., yes, or no). Third, fisher’s exact test was used to examine the association between participants’ demographics (i.e., professional rank, years of experience) and participation in research (i.e., yes, or no), and the association between participants’ demographics (i.e., gender, professional rank) and number of publications (i.e., once, or twice), all analyses were conducted utilizing the JMP® Software (JMP®, Version 16. SAS Institute Inc., Cary, NC, 1989–2023), while using a statistical significance level of 0.05.

## Results

### Demographic characteristics

Among the 137 radiology practitioners and interns who were invited to participate, 112 responded and completed the questionnaire, resulting in an overall response rate of 81.75%. Participants’ demographics are shown in Table 1. The median age of the participants was 25 years, and the mean age was 27.6 ± 7.2 years (range, 22–52 years). Among the 112 radiology practitioners and interns participating in this study, 68 (60.7%) were male, and 44 (39.3%) were female, 109 (97.3%) were Saudis, and 3 (2.7%) were from other nationalities, 82 (73.2%) were single and 30 (26.8%) were married, and 55 (49.1%) were from Jeddah city, 39 (34.8%) from Riyadh, and 18 (16.1%) from Al-Ahsa. A total of 11 (9.82%) had a GPA of ≤3.00–3.50, while 36 (32.1%) had 3.51–4.00, 37 (33%) had 4.01–4.50, 25 (22.3%) had 4.50–5.00, and 3 (2.68%) preferred not to disclose their GPAs. The sample comprised of participants from a variety of imaging departments. Of the total, 49 (43.7%) radiology practitioners and interns worked in the Radiography/Mammography/Fluoroscopy departments, 17 (15.2%) were from MRI, 17 (15.2%) from CT, 2 (1.8%) from Nuclear Medicine, 19 (17%) belonged to Ultrasonography, and 8 (7.1%) were from Interventional Radiology departments. Additionally, the sample included 59 (52.7%) Technologists or Specialists, 15 (13.4%) Senior Specialists, 36 (32.1%) Radiology Interns, and 2 (1.79%) Technicians. A total of 56 (50%) participants had less than 1 year of experience, 15 (13.4%) had 1–3 years of experience, 16 (14.3%) had 3–5 years of experience, and 25 (22.3%) had more than 5 years of experience. Most study participants (n = 108, 96.4%) obtained their radiological degrees in Saudi Arabia, while the remaining (n = 4, 3.6%) obtained them outside the country.

**Table 1:** KAMCs’ radiology practitioners and interns’ demographic characteristics.

Variables		Total Sample (*n* = 112)
Age in years “Median, mean ± standard deviation, (range)”		“25, 27.6 ± 7.2, (22–52)”
Gender “*n* (%)”	Male	68 (60.7%)
	Female	44 (39.3%)
Nationality “*n* (%)”	Saudi	109 (97.3%)
	Others	3 (2.7%)
Marital status “*n* (%)”	Single	82 (73.2%)
	Married	30 (26.8%)
Grade point average (GPA) “*n* (%)”	≤3.00–3.50	11 (9.82%)
	3.51–4.00	36 (32.1%)
	4.01–4.50	37 (33%)
	4.50–5.00	25 (22.3%)
	Prefer not to tell	3 (2.68%)
King Abdulaziz Medical Cities (KAMCs) Location “*n* (%)”	Jeddah City	55 (49.1%)
	Riyadh City	39 (34.8%)
	Al-Ahsa City	18 (16.1%)
Medical imaging division/subspeciality “*n* (%)”	Radiography/mammography/fluoroscopy	49 (43.7%)
	Magnetic resonance imaging (MRI)	17 (15.2%)
	Computed tomography (CT)	17 (15.2%)
	Nuclear medicine	2 (1.8%)
	Ultrasonography	19 (17%)
	Interventional radiology	8 (7.1%)
Professional rank “*n* (%)”	Technician	2 (1.79%)
	Technologist or specialist	59 (52.7%)
	Senior specialist	15 (13.4%)
	Radiology intern	36 (32.1%)
Years of Experience “*n* (%)”	Less than 1 year	56 (50%)
	1–3 years	15 (13.4%)
	3–5 years	16 (14.3%)
	More than 5 years	25 (22.3%)
Country that you obtain your radiological technologist degree from “*n* (%)”	Saudi Arabia	108 (96.4%)
	Others	4 (3.57%)

KAMCs, King Abdulaziz Medical Cities.

$\mathrm{Percentage}\;\mathrm{of}\;\mathrm{Responses}\;\left(\%\right)=\frac{\mathrm{Number}\;\mathrm{of}\;\mathrm{Responses}}{112}\times 100$ .

### Obstacles or barriers preventing research pursuits

Table 2 shows the obstacles or barriers that may impede the radiology practitioners’ and interns’ ability to conduct research. The potential personal-related reasons for not conducting research encompassed lack of interest (*n* = 44, 39.3%), deficiency in fundamental research skills (*n* = 51, 45.5%), inadequate research facilities (*n* = 29, 25.9%), and personal commitments such as family, problems, marriage (*n* = 43, 38.4%), whereas 11 (9.82%) do not acknowledge the importance of research. On the contrary, reasons for not conducting research from an institutional perspective included a lack of interest among faculty (*n* = 45, 40.2%), lack of professional supervisor support (*n* = 57, 50.9%), lack of research curriculum (*n* = 19, 17%), inadequate financial support (*n* = 36, 32.1%), lack of time (*n* = 74, 66.1%), a complicated research approval process (*n* = 25, 22.3%), a shortage of patients (*n* = 7, 6.25%), and difficulty in challenges in patient follow-up (*n* = 21, 18.8%).

**Table 2:** Obstacles or barriers preventing 112 KAMCs’ radiology practitioners and interns from conducting research.

Obstacles	Total sample (*n* = 112)
	Responses *n* (%)
**Personal reasons**	
Lack of interest	44 (39.3%)
Lack of baseline research skills	51 (45.5%)
Inadequate facilities for research	29 (25.9%)
Personal commitments like family, problems, marriage	43 (38.4%)
You do not believe in its importance	11 (9.82%)
**Institutional reasons**	
Lack of interest by faculty	45 (40.2%)
Lack of professional supervisor support	57 (50.9%)
Lack of research curriculum	19 (17%)
Inadequate financial support	36 (32.1%)
Lack of time	74 (66.1%)
Process of research approval is complicated	25 (22.3%)
Insufficient number of patients	7 (6.25%)
Difficulty in following up with patients	21 (18.8%)

KAMCs, King Abdulaziz Medical Cities.

$\mathrm{Percentage}\;\mathrm{of}\;\mathrm{Responses}\;\left(\%\right)=\frac{\mathrm{Number}\;\mathrm{of}\;\mathrm{Responses}}{112}\times 100$ .

### Involvement in research work

**Figure 1:**
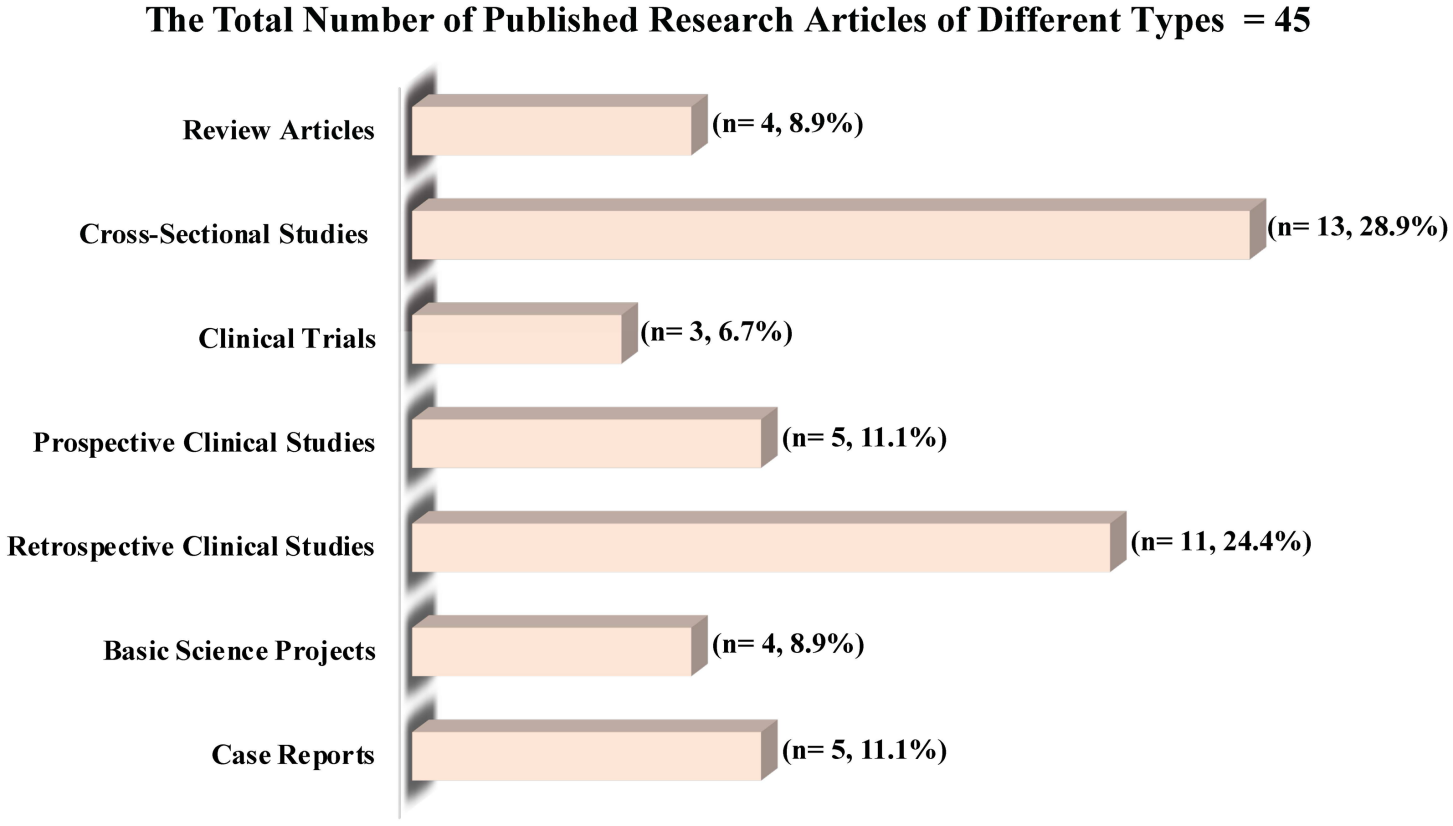
Type of research articles published by the 38 KAMCs’ radiology practitioners and interns who participated in a research.

Table 3 shows the association between KAMCs’ radiology practitioners and interns’ demographics and their participation in research. Of the 112 KAMCs’ radiology practitioners and interns, 83% or 93 participants were engaged in research work. KAMCs practitioners and interns’ demographics, including gender, marital status, professional rank, years of experience, and medical imaging division/subspeciality, did not demonstrate a significant association with their participation in research (*p* > 0.05). The participation rate in research was similar for both males (83.8%) and females (81.8%), as well as for those who were single (86.6%) and those who were married (73.3%). Additionally, similar research participation rates were found among Technologists or Specialists (83%), Interns (86.1%), and Senior Specialists (80%), while between the two participating Technicians, one (50%) participated in research. Similar rates of participation in research were also found, regardless of variations in years of experience. For instance, individuals with less than 1 year of experience had a participation rate of 87.5%, while those with 1–3 years of experience had a participation rate of 80%. Those with 3–5 years of experience had a participation rate of 87.5%, and those with more than 5 years of experience had a participation rate of 72%. The rates of participation in research across various medical imaging divisions/subspecialties were 73.5% for Radiography/Mammography/Fluoroscopy, 88.2% for MRI, 82.4% for CT, 100% for nuclear medicine, 94.7% for ultrasonography, and 100% for interventional radiology. Table 4 shows the association between KAMCs’ radiology practitioners and interns’ demographics and number of publications. Of the 93 KAMCs’ radiology practitioners and interns who took part in radiologic research, 40.9% or 38 participants were able to successfully publish a total of 45 research articles. Of the 38 participants who published their research, 31 (81.6%) had one publication and 7 (18.4%) had two publications. KAMCs practitioners and interns’ demographics, including gender, and professional rank, did not demonstrate a significant association with the number of publications (*p* > 0.05). Figure 1 presents the various types of research articles that were published (*n* = 45), including cross-sectional studies (*n* = 13, 28.9%), retrospective clinical studies (*n* = 11, 24.4%), prospective clinical studies (*n* = 5, 11.1%), case reports (*n* = 5, 11.1%), review articles (*n* = 4, 8.9%), basic science projects (*n* = 4, 8.9%), and clinical trials (*n* = 3, 6.7%).

**Table 3:** Association between KAMCs’ radiology practitioners and interns’ demographics and their participation in research.

Variable	Participation in research *n* (%)		*p*-value
	Yes	No	
**Gender**			
Male *(n = 68)*	57 (83.8%)	11 (16.2%)	0.7824^a^
Female *(n = 44)*	36 (81.8%)	8 (18.2%)	
**Marital status**			
Single *(n = 82)*	71 (86.6%)	11 (13.4%)	0.0980^a^
Married *(n = 30)*	22 (73.3%)	8 (26.7%)	
**Professional rank**			
Technician *(n = 2)*	1 (50%)	1 (50%)	0.4439^b^
Technologist or specialist *(n = 59)*	49 (83%)	10 (17%)	
Senior specialist *(n = 15)*	12 (80%)	3 (20%)	
Radiology intern *(n = 36)*	31 (86.1%)	5 (13.9%)	
**Years of experience**			
Less than 1 year *(n = 56)*	49 (87.5%)	7 (12.5%)	0.3414^b^
1–3 years *(n = 15)*	12 (80%)	3 (20%)	
3–5 years *(n = 16)*	14 (87.5%)	2 (12.5%)	
More than 5 years *(n = 25)*	18 (72%)	7 (28%)	
**Medical imaging division/subspeciality**			
Radiography/mammography/fluoroscopy *(n = 49)*	36 (73.5%)	13 (26.5%)	0.2590^b^
Ultrasonography *(n = 19)*	18 (94.7%)	1 (5.3%)	
Magnetic resonance imaging (MRI; *n = 17*)	15 (88.2%)	2 (11.8%)	
Computed tomography (CT; *n = 17*)	14 (82.4%)	3 (17.6%)	
Interventional radiology *(n = 8)*	8 (100%)	0 (0%)	
Nuclear medicine *(n = 2)*	2 (100%)	0 (0%)	

KAMCs, King Abdulaziz Medical Cities. ^a^Chi-square test used. ^b^Fisher’s exact test used.

$\begin{array}{l} \mathrm{Percentage}\;\mathrm{of}\;\mathrm{Responses}\;\left(\%\right)\\ =\frac{\mathrm{Number}\;\mathrm{of}\;\mathrm{Responses}}{\mathrm{Total}\;\mathrm{Number}\;\mathrm{of}\;\mathrm{sample}\;\mathrm{in}\;\mathrm{Each}\;\mathrm{Demographical}\;\mathrm{Variable}}\times 100 \end{array}$ .

**Table 4:** Association between KAMCs’ radiology practitioners and interns’ demographics and number of publications.

Variable	Number of participants who published their research = 38		*p*-value
	Once (*n* = 31)	Twice (*n* = 7)	
**Gender**			
Male	19 (61.3%)	5 (71.4%)	0.6155^a^
Female	12 (38.7%)	2 (28.6%)	
**Professional rank**			
Technologist or specialist	19 (61.4%)	5 (71.4%)	0.5968^b^
Senior specialist	6 (19.3%)	2 (28.6%)	
Radiology intern	6 (19.3%)	0 (0%)	

KAMCs, King Abdulaziz Medical Cities. ^a^Chi-square test used. ^b^Fisher’s exact test used.

$\mathrm{Percentage}\;\mathrm{of}\;\mathrm{Responses}\;\left(\%\right)=\frac{\mathrm{Number}\;\mathrm{of}\;\mathrm{Responses}}{38}\times 100$ .

### Perceptions of the importance of research

**Figure 2:**
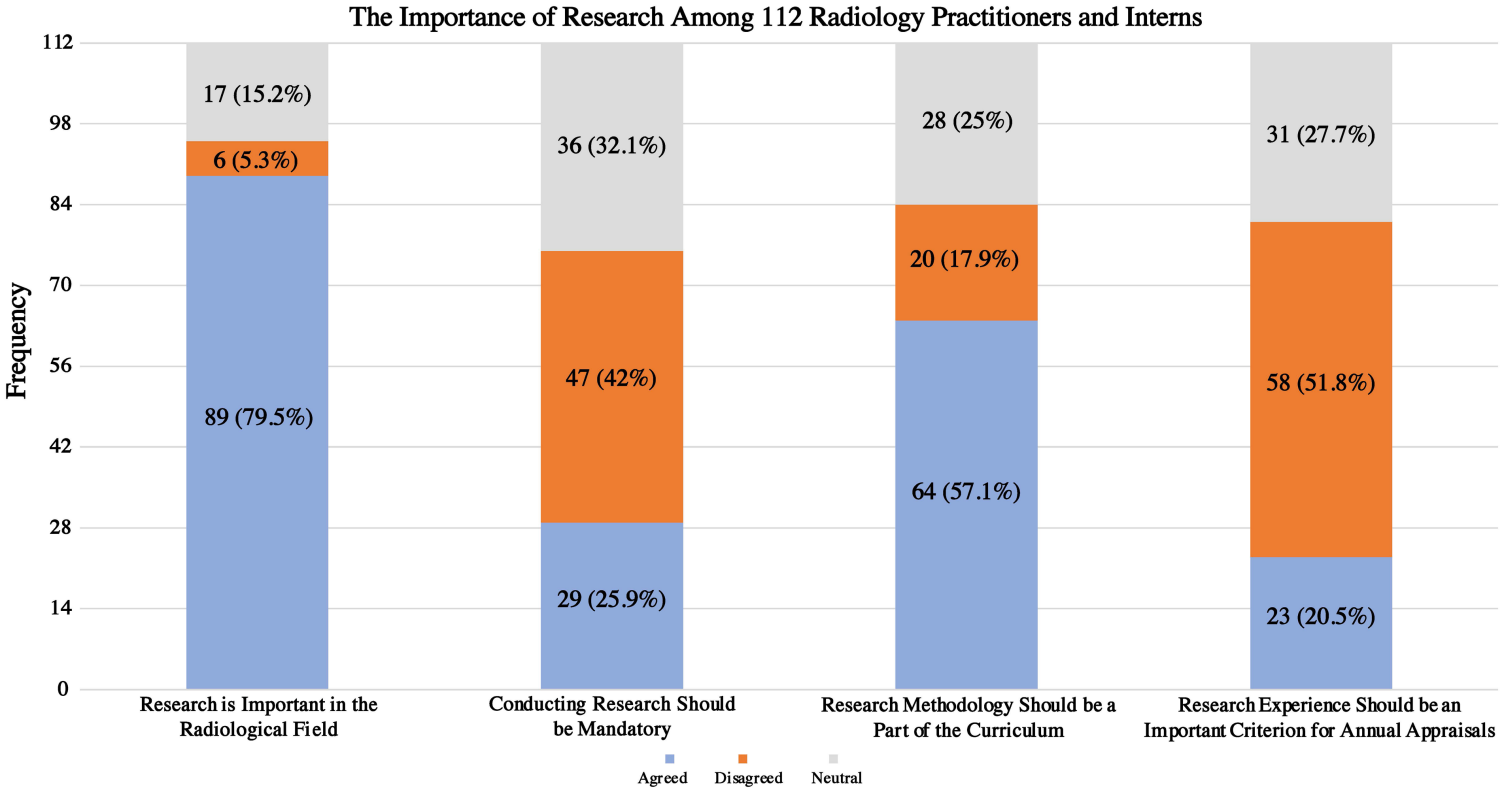
The perception of KAMCs’ radiology practitioners and interns of research’s importance and impact on their careers.

Figure 2 displays the viewpoints of 112 radiology practitioners and interns regarding the importance of research and its potential influence on their career growth. The majority of respondents (*n* = 89, 79.5%) agreed that radiologic research is important, while 6 (5.3%) disagreed, and 17 (15.2%) were neutral. Additionally, only 29 (25.9%) agreed that conducting research should be mandatory, while 47 (42%) disagreed, and 36 (32.1%) were neutral. Most respondents (*n* = 64, 57.1%) agreed that research methodology should be part of the curriculum, while 20 (17.9%) disagreed, and 28 (25%) were neutral. Furthermore, only 23 (20.5%) agreed that research experience should be an important criterion for annual appraisals, while 58 (51.8%) disagreed and 31 (27.7%) were neutral.

### Motives to conduct research

**Figure 3:**
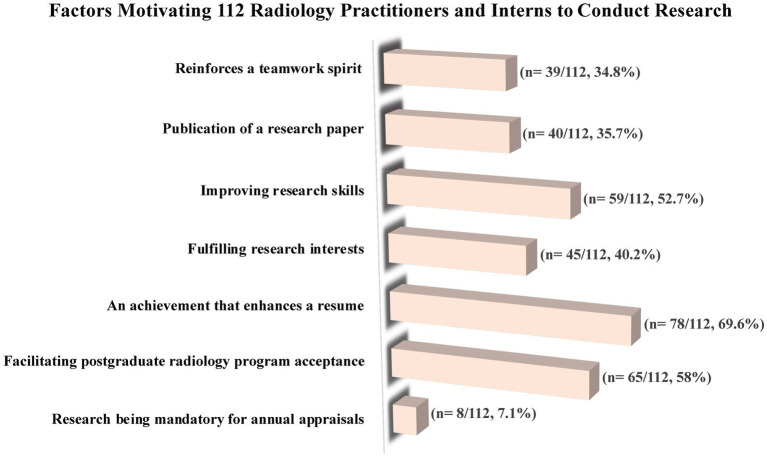
The factors motivating KAMCs’ radiology practitioners and interns to conduct research.

Figure 3 depicts the underlying seven motivations that drove 112 radiology practitioners and interns to conduct research. The most prevalent motive among participants (*n* = 87, 69.6%) was the desire to enhance their resumes through the accomplishment of radiologic research. This was followed by the aspiration to facilitate acceptance into postgraduate radiology programs (*n* = 65, 58%), the goal of improving research skills (*n* = 59, 52.7%), the fulfillment of research interests (*n* = 45, 40.2%), the publication of a research paper (*n* = 40, 35.7%), the reinforcement of a teamwork spirit through research (*n* = 39, 34.8%), and the mandatory nature of research for annual appraisals (*n* = 8, 7.1%).

## Discussion

This study provides valuable insights into the opinions of radiology practitioners and interns in Saudi Arabia regarding radiologic research. To our knowledge, this is the first study to assess the perceptions and attitudes of radiology practitioners and interns in Saudi Arabia toward research and to explore the various barriers and obstacles that hinder their research efforts. In this study, radiology practitioners and interns from various medical imaging subspecialties were found to be involved in research to the extent of 83%, with nearly half (40.9%) of them have had publications, and 53.3% of these publications being either cross-sectional studies or retrospective clinical studies. A lack of time, and a lack of a professional supervisor support program were common obstacles that may impede the radiology practitioners’ and interns’ ability to conduct research. The Importance of radiologic research is well recognized by 79.5% of the participants, and with 57.1% believe that research methodology ought to be an integral component of the radiologic curriculum. Additionally, the most common motives for radiology practitioners and interns to conduct research were the desire to improve their resumes (69.6%), get accepted into postgraduate radiology programs (58%), and improve their research skills (52.7%).

Although a large majority of the participants (83%) in our study participated in radiologic research, the specifics of their contributions were not identified. Radiographers have been reported to take part in a variety of research-related activities, with data collection being the primary focus, which suggests that radiographers are not heavily engaged in active research pursuits (2, 30, 33). Even though radiographers may be involved in the collection of clinical data, their names are not usually included in published articles. The reason for this is that radiographers are not typically recognized as members of the academic research team because they do not play a significant role in the conception of research ideas, research design, execution, data analysis, or manuscript writing for academic research (1, 2). Being engaged and participating in a research group can offer exceptional learning prospects, facilitate the enhancement of research abilities, establish a valuable network with fellow researchers, and foster confidence in one’s professional life (1, 2). Furthermore, engaging in research requires establishing a thriving radiography research culture within the workplace (1).

A lack of time to conduct research was identified as the primary hindrance (66.1%) to the involvement of KAMCs’ radiology practitioners and interns in research, which was consistent with previous studies conducted among radiographers (31, 32, 39), physicians (35, 37, 40, 41), nursing professionals (42, 43), and medical students (36, 44). Managing the fast-paced healthcare environment within the diagnostic imaging field and making valuable contributions to radiologic research may seem daunting, but it is certainly achievable (39). It is crucial to adopt a positive outlook toward EBP to recognize that research is not only essential for providing high-quality healthcare but also for improving workflow (1, 31). To foster a research culture in radiology, it is imperative to educate radiology practitioners on the significance of EBP and inspire them to embrace this mindset shift (45). This approach is expected to enhance the overall research culture within the radiology field (1, 45).

Nearly half of the participants (50.9%) in this study have also identified the absence of professional supervisor support as an obstacle to their research involvement. It is noteworthy that the support of colleagues and other professionals has been identified as a crucial enabler/facilitator for radiography research (1, 46). A lack of research skills along with a lack of interest were also identified as barriers to conducting research by more than 40% of KAMCs’ radiology practitioners and interns. This finding is quite similar to that of studies conducted previously among radiographers in the Nordic countries (1), Singapore (32), and Saudi medical and surgical residents (37), This emphasizes the importance of creating a research culture within the radiology profession. A lack of a research culture has previously been reported in radiography (47). A culture needs to be defined in terms of its context, as well as how it may affect the possibility of change (5, 48). Additionally, a culture is comprised of “attitudes, norms, and values,” while a research culture pertains to research activities and the essential requirements. For instance, the “attitude” of radiographers toward recognizing their peers’ contributions in conducting research, the “norm” of research collaboration among professionals within and across fields, and “valuing” the importance of EBP in the workplace (1, 2).

Similarly to previous studies (2, 30–33, 41), the participants in this study expressed positive attitudes toward radiologic research. Although a significant proportion of KAMCs’ radiology practitioners and interns (79.5%) strongly recognize the importance of radiologic research, and 57.1% believe that research methodology should be an essential part of the radiology curriculum, nearly half of them (47%) do not agree that conducting research should be obligatory, and also nearly half of the study participants (51.8%) do not consider research experience as a crucial criterion for annual appraisals. Despite the positive attitude displayed, there was a difference in terms of actual participation and actual publication of research as only 40.9% of KAMCs’ radiology practitioners and interns who stated that they participated in research actually published their research. Similar results were found among clinical and academic post graduate doctors (49), physicians (37, 40), residents (50), and medical students (51). However, radiologic research publications among KAMCs’ students and faculty have increased in recent years. For instance, during the year of 2018–2019, three research articles were published (52–54), followed by a lack of publications in 2020. Yet, in 2021, there was a considerable rise with seven research publications (55–61), and it continued to grow with 10 research publications in 2022–2023 (62–71).

Herein, the participation in research or number of publications among KAMCs’ participants was not significantly associated with their demographic characteristics, including gender, marital status, professional rank, years of experience, and medical imaging division/subspecialty. In other words, similar rates of participation in research were found across KAMCs’ demographic data. On the contrary, previous studies have shown that married residents and medical students tend to participate in research more frequently than their single counterparts (36, 37). Furthermore, the same studies indicated that residents who have graduated with GPAs ranging from 3 to 3.74 and 3.75 to 4.24 are more likely to participate in research than those with lower (<3) or higher (>0.4.25) GPAs. Additionally, it has been found that junior residents demonstrate a higher level of participation in research when compared to senior residents (36, 37).

In our study, the most common motives among participants to conduct research were the desire to enhance their resumes through the accomplishment of radiologic research (69.6%), the aspiration to facilitate admission into postgraduate radiology programs (58%), and the aim of improving research skills (52.7%). In contrast, senior medical students in Saudi Arabia reported even higher percentages of these motives, indicating that they have a greater motivation to conduct research than KAMCs’ radiology practitioners and interns (36).

### Strengths and limitations and future research

One of the main strengths of this study is the anonymity of radiology practitioners and interns working in three hospitals; therefore, our findings reflect their genuine perceptions of this subject, free from the potential influence of personal interview bias, which can alter their opinions. The questionnaire was validated previously, which makes our findings more reliable. Additionally, our study can serve as a pilot study for future research into this specific aspect of radiology practitioners’ and interns’ professional development. The limitations of this study stem from the use of non-probability convenience sampling method, which limits the generalizability of the results to all radiology practitioners and interns in Saudi Arabia and other countries. As this study utilized a validated questionnaire and a quantitative approach, we could not obtain any qualitative insights from the radiology practitioners and interns that could have been valuable. Although 83% of our study participants took part in radiologic research, the nature of their contribution was not investigated in our study. This information would have been valuable in assessing their research needs, strengths, and weaknesses, ultimately leading to better planning of research training and development programs. Therefore, future research should include a large sample size of radiology practitioners and interns from multiple Saudi Arabian health centers and evaluate the nature of their contributions to research as well as conduct qualitative analyses of motivations and perceived barriers.

## Conclusion

Research plays a vital role in the field of radiologic technology and is an essential component of EBP. It is imperative to engage radiology practitioners and interns in radiologic research and encourage radiology practitioner-led research, which are both crucial steps in advancing the profession’s evidence base and adopting new clinical practices. Our findings show that, KAMCs’ radiology practitioners and interns have a positive attitude toward performing research and they consider it an integral component of the radiology profession. Despite the high percentage (83%) of those involved in research, the number of publications remains low. The primary motives for radiology practitioners and interns to conduct research were enhancing their resumes, gaining admission to postgraduate radiology programs, and improving their research skills. In contrast, the primary obstacles were the lack of time, lack of a professional supervisor support program, lack of research skills. Hence, healthcare administrators should provide radiology practitioners and interns with the necessary resources and support to enhance their research capabilities, including time, technical tools, and research methodology workshops. These findings can serve as a valuable basis for designing developmental programs aimed at overcoming these obstacles.

## Data availability statement

The raw data supporting the conclusions of this article will be made available by the authors, without undue reservation.

## Ethics statement

King Abdullah International Medical Research Center Ethics Committee approved this study (Study Number: SP22J/099/08). We confirm that a written informed consent was obtained from the study participants and that the guidelines outlined in the Declaration of Helsinki were followed.

## Author contributions

KA: Conceptualization, Data curation, Formal Analysis, Investigation, Methodology, Project administration, Resources, Software, Supervision, Validation, Visualization, Writing – original draft, Writing – review & editing. AA: Conceptualization, Resources, Supervision, Writing – review & editing. RK: Investigation, Resources, Supervision, Writing – review & editing. KA: Data curation, Formal Analysis, Investigation, Methodology, Project administration, Software, Writing – original draft. WM: Data curation, Formal Analysis, Investigation, Methodology, Project administration, Software, Writing – original draft. AA: Data curation, Formal Analysis, Investigation, Methodology, Project administration, Software, Writing – original draft. FA: Data curation, Formal Analysis, Investigation, Methodology, Project administration, Software, Writing – original draft. HA: Data curation, Formal Analysis, Software, Writing – review & editing. EG: Data curation, Formal Analysis, Software, Writing – review & editing. ST: Investigation, Project administration, Supervision, Writing – review & editing. AA: Investigation, Project administration, Supervision, Writing – review & editing.
